# Ultrafast laser state active controlling based on anisotropic quasi-1D material

**DOI:** 10.1038/s41377-024-01423-3

**Published:** 2024-04-07

**Authors:** Zixin Yang, Qiang Yu, Jian Wu, Haiqin Deng, Yan Zhang, Wenchao Wang, Tianhao Xian, Luyi Huang, Junrong Zhang, Shuai Yuan, Jinyong Leng, Li Zhan, Zongfu Jiang, Junyong Wang, Kai Zhang, Pu Zhou

**Affiliations:** 1 College of Advanced Interdisciplinary Studies, National University of Defense Technology, Changsha, 410073 China; 2 Nanhu Laser Laboratory, National University of Defense Technology, Changsha, 410073 China; 3 i-Lab & Key Laboratory of Nanodevices and Applications & Key Laboratory of Nanophotonic Materials and Devices, Suzhou Institute of Nano-Tech and Nano-Bionics, Chinese Academy of Sciences, Suzhou, 215123 China; 4 School of Applied and Engineering Physics, Cornell University, Ithaca, NY 14853 USA; 5 State Key Laboratory of Advanced Optical Communication Systems and Networks, School of Physics and Astronomy, Shanghai Jiao Tong University, Shanghai, 200240 China; 6 Shanghai Key Lab of Modern Optical System, University of Shanghai for Science and Technology, Shanghai, 200093 China

**Keywords:** Ultrafast lasers, Nanowires

## Abstract

Laser state active controlling is challenging under the influence of inherent loss and other nonlinear effects in ultrafast systems. Seeking an extension of degree of freedom in optical devices based on low-dimensional materials may be a way forward. Herein, the anisotropic quasi-one-dimensional layered material Ta_2_PdS_6_ was utilized as a saturable absorber to modulate the nonlinear parameters effectively in an ultrafast system by polarization-dependent absorption. The polarization-sensitive nonlinear optical response facilitates the Ta_2_PdS_6_-based mode-lock laser to sustain two types of laser states, i.e., conventional soliton and noise-like pulse. The laser state was switchable in the single fiber laser with a mechanism revealed by numerical simulation. Digital coding was further demonstrated in this platform by employing the laser as a codable light source. This work proposed an approach for ultrafast laser state active controlling with low-dimensional material, which offers a new avenue for constructing tunable on-fiber devices.

## Introduction

Ultrafast fiber lasers are becoming more popular as bulk laser alternatives, with applications in micromachining^1–3^, biomedicine^4,5^, ultrafast physics^6–9^, multiphoton microscopy^10–12^, and spectroscopy^13,14^. Many innovations and applications require tunable ultrafast laser parameters, including wavelength, intensity, pulse width, laser state (LS), etc^15^. Due to complex nonlinear effects within the ultrafast system, it is still a great challenge for laser state active controlling (LSAC) of ultrafast fiber lasers. LSAC has been reported in nonlinear ultrafast systems such as traditional nonlinear polarization rotation (NPR) by adjusting the polarization controller (PC) to change the intracavity parameters. However, this approach has limitations in modulation bandwidth and environmental stability^16,17^. A fundamental way to achieve stable LSAC is to increase the tunability of fiber-based devices. Low-dimensional materials (LDMs) with the quantum confinement effect exhibit unique optoelectronic properties, providing new opportunities for generation of ultrafast lasers^18–27^. The ultrafast lasers based on LDMs exhibits decent performance in pulse width and spectra^28–31^ while the LS controlling is elusive. Even though LS controlling can be achieved by regulating pump power^32–34^ or thickness of saturable absorber (SA)^35^, it typically suffers from low effectiveness and lack of dynamic mechanism.

LDMs with anisotropic crystal structure provide an additional degree of freedom for light modulation by polarization control, offering opportunity for tunable optoelectronic devices^36^. Recently, anisotropic LDMs have been harnessed in polarization-sensitive optoelectronic devices such as photodetectors^37,38^ and polarized light-emitting diodes^39,40^. However, their application in ultrafast photonics has yet to be reported by exploiting their prominent polarization-sensitive absorption. Layered materials with broadband photoresponse and high air stability are required for their application in ultrafast photonics. As a ternary anisotropic van der Waals material, Ta_2_PdS_6_ features high air stability because of strong d^2^sp^3^ hybridization between chalcogen atoms and group-10 precious elements^41^. Furthermore, its band gap varies from nearly 0 eV to 1.0 eV with different thicknesses, resulting in broadband photoresponse in optoelectronic applications^18^.

**Fig. 1:**
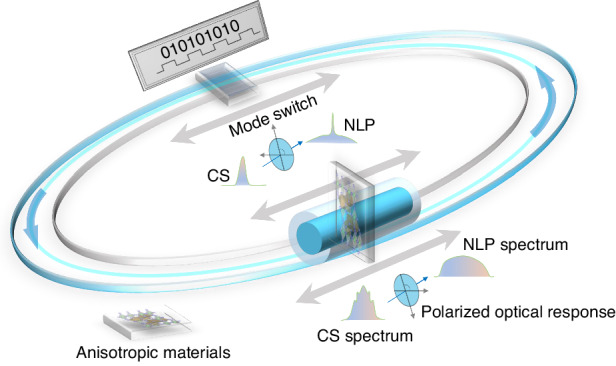
LSAC ultrafast dynamics platform based on anisotropic “0−1”-switcher. Active controlling of the laser state of ultrafast systems using the polarized optical response of anisotropic materials, and its potential applications in digital coding. CS conventional soliton, NLP noise-like pulse

In this work, the stable LSAC ultrafast dynamics platform is demonstrated by using a high-quality quasi-one-dimensional (1D) Ta_2_PdS_6_ “0−1”-switcher, as shown in Fig. 1. The twin-detector experiment reveal that the quasi-1D anisotropic Ta_2_PdS_6_ presents distinct nonlinear polarization optical response. A “0−1”-switcher performing steady LSAC between conventional soliton (CS) and noise-like pulse (NLP) has been achieved based on polarization control. The numerical simulation elucidates the dynamical processes of CS (“0”) and NLP (“1”) states. The digital coding applicability has been further demonstrated in this platform. These findings pave the way for tunable ultrafast laser devices based on anisotropic LDMs.

## Results

### Polarization-dependent optical response in anisotropic layered Ta_2_PdS_6_

**Fig. 2:**
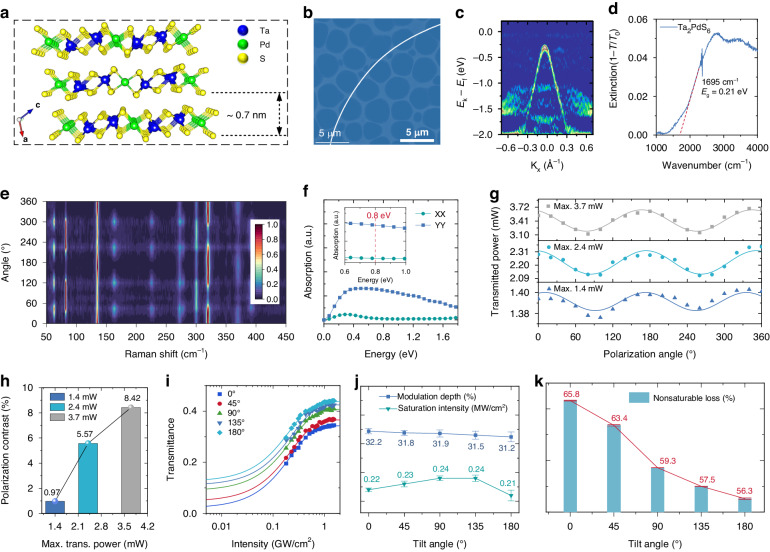
The anisotropic structure, scattering, and polarization-dependent optical response of the quasi-1D Ta_2_PdS_6_. **a** The crystal structure of Ta_2_PdS_6_ observed along the b-axis. **b** Low-magnification TEM image of quasi-1D Ta_2_PdS_6_. **c** ARPES measurement of the Ta_2_PdS_6_ crystal. **d** FTIR spectrum of the Ta_2_PdS_6_. **e** The polarized Raman spectra of Ta_2_PdS_6_. **f** The direction-dependent optical properties of Ta_2_PdS_6_. **g** The polarization-dependent transmission at various incidence powers. **h** The polarization contrast at various maximum transmitted powers. **i** Ta_2_PdS_6_ nonlinear transmission versus energy intensity at various tilt angles. **j** The saturation intensity and modulation depth of Ta_2_PdS_6_ for various tilt angles. **k** The nonsaturable loss of Ta_2_PdS_6_ for various tilt angles

Ta_2_PdS_6_ was a stable layered material with reduced in-plane symmetry (Fig. 2a)^18^. Single-crystal Ta_2_PdS_6_ were synthesized via a chemical vapor transport (CVT) approach (see Methods and Fig. S1a in Supporting Information) and the analysis of the elements together with microscopic morphology demonstrated the expected atomic ratio and high quality (Figs. S1 and S2, Supporting Information). The obtained crystals and flakes typically appear needle-like morphology, manifesting their quasi-1D structure (Fig. 2b and Fig. S1b in Supporting Information). High-resolution transmission electron microscopy in Fig. S1c (Supporting Information) further reveals the microscopic structure anisotropy. As demonstrated by angle-resolved photoemission spectroscopy (ARPES) and Fourier transform infrared (FTIR) absorption spectrum in Fig. 2c, d, Ta_2_PdS_6_ has a narrow bandgap of ~0.21 eV, rendering its wide band absorption in the infrared regime.

Anisotropic optical responses were expected from the reduced in-plane symmetry. Polarized Raman spectra of Ta_2_PdS_6_ flake (Fig. 2e and Fig. S3 in Supporting Information) show anisotropic Raman intensities corresponding to the typical ${A}_{g}$ and ${B}_{g}$ vibrational modes^42^, indicating the polarization-sensitive phonon vibrations. The UV-Vis-NIR spectrum in Fig. S4 (Supporting Information) reveals the broad-band absorption from Ta_2_PdS_6_ with approximately 62% at 1560 nm, which is chosen in the subsequent pulsed laser experiments. The polarized optical absorption of the Ta_2_PdS_6_ was first theoretically simulated (See Methods)^43^. The results in Fig. 2f illustrate a prominent polarization-dependent absorption characteristic of Ta_2_PdS_6_ at 1.56 μm (corresponding to about 0.8 eV). The anisotropic transmittance of Ta_2_PdS_6_ was measured experimentally with a wavelength of 1.56 μm (see Methods and Fig. S5a in Supporting Information). The polarization-dependent transmitted power for Ta_2_PdS_6_ changes under incident powers of 3.9 mW, 6.7 mW, and 10.5 mW, respectively, as shown in Fig. 2g. The polarization-dependent absorption properties of Ta_2_PdS_6_ exhibited different maximum transmitted powers of 1.4 mW, 2.4 mW, and 3.7 mW, respectively. The transmittance is sensitive to the polarization of the incident light and the polarization contrasts increase with pump power (Fig. 2h), which offers an extra degree of freedom to regulate pulsed laser performance.

The polarization-dependent saturable absorption of Ta_2_PdS_6_ was further investigated by the all-fiber twin-detector configuration (see Methods and Fig. S5b in Supporting Information). In the experiment, the light polarization was altered by tilting the angle of the PC from 0° to 180° with a step of 45°. We note that the rotation angle of the PC does not directly reflect the direction of the laser polarization state. Nevertheless, the large tilt angles offer a convenient way to change the optical polarization in the following ultrafast laser experiments. As shown in Fig. 2i, the nonlinear transmittance differs with the tilt angles of PC. The key parameters of SA were fitted by

1 $$T(I)=1-\frac{\varDelta R}{1+I/{I}_{s}}-{T}_{ns}$$

where the $T(I)$ is the transmittance rate, $I$, $\varDelta R$, ${I}_{s}$, and ${T}_{ns}$ are the input intensity, the modulation depth, saturation intensity, and nonsaturable loss, respectively. As shown in Fig. 2j, both the saturation intensity and the modulation depth depend on the tilt angle. The high modulation depth (over 31%) of Ta_2_PdS_6_, benefits its application as SA for ultrafast pulse generation. Specifically, the nonsaturable loss exhibits a significant variation with the maximum saturation absorption loss of about 65.8% at the tilt angle of 0° and the minimum saturation loss of about 56.3% at the tilt angle of 180° (Fig. 2k). Since the saturable absorption results from the Pauli blocking principle^7^ (Fig. S6, Supporting Information), the polarization-dependent nonsaturable loss variation in the quasi-1D layered materials offers new opportunities for regulating nonlinear absorption behavior.

### Ultrafast fiber lasers with CS and NLP states

**Fig. 3:**
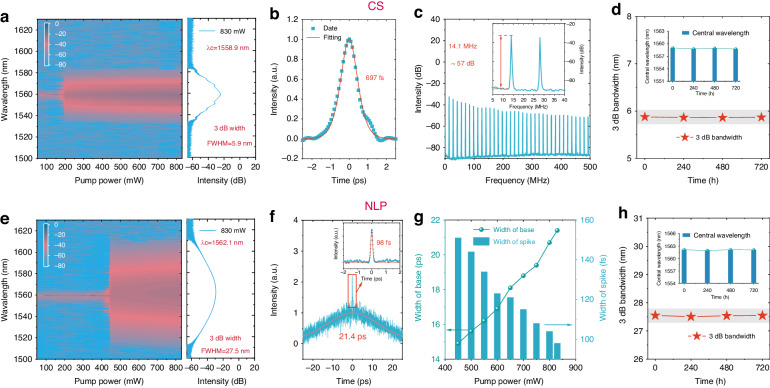
Spectrum and frequency domain of the CS and NLP lasers based on Ta_2_PdS_6_. **a** Spectral evolution under different pump power and optical spectrum of CS at 830 mW. **b** Autocorrelation trace. **c** The RF spectrum of the CS state laser. **d** The central wavelength and 3 dB bandwidth of CS spectra obtained at various time durations. **e** Spectral evolution under different pump power and optical spectrum of NLP at 830 mW. **f** Autocorrelation trace of NLP state laser at 830 mW. **g** NLP base and spike width versus pump power. **h** The central wavelength and 3 dB bandwidth of NLP spectra obtained at various time durations

Ta_2_PdS_6_-based ultrafast fiber lasers were constructed by integrating Ta_2_PdS_6_ precisely in the center of the fiber end face by a fully dry transfer process (see Methods and Fig. S5c in Supporting Information). Atomic force microscopy measurements indicate that the thickness of the Ta_2_PdS_6_ is about 40 nm with good flatness. To verify its ability to generate ultrashort pulses, the Ta_2_PdS_6_ was removed from the ring cavity or a linear polarizer was utilized in place of the Ta_2_PdS_6_, and no ultrafast pulse output phenomenon was observed by adjusting the state of the PC and the pump power values. Firstly, the CS LSs were successfully generated in the Ta_2_PdS_6_-based fiber laser. The threshold pump power from continuous wave to CS state was 200 mW, as unambiguously demonstrated by the change of the optical spectrum with the pumping power (Fig. 3a). The laser can supply steady CS pulses within the pump power range of 200−830 mW and the maximum average output power was 37 mW with a corresponding slope efficiency of about 4.5% (Fig. S7a, Supporting Information). The spectrum of CS mode was centered at 1558.9 nm with a 3 dB bandwidth of 5.9 nm (Fig. 3a). The pulse-to-pulse separation of 70.9 ns and repetition frequency of 14.1 MHz (Fig. S7b, Supporting Information) match well with the cavity length, indicating that the fiber laser works in a mode-locked state. A pulse duration of 697 fs was extracted from the autocorrelation trace data (Fig. 3b), further corroborating the laser working in the typical conventional soliton regime. The spike of the basic repetition frequency was located at 14.1 MHz without side peaks, and a signal-to-noise ratio (SNR) was about 57 dB measured by the radio frequency (RF) spectrum, indicating high stability of CS operation (Fig. 3c). The laser under CS mode was further recorded for more than 720 h under ambient conditions with fixed experimental conditions and no significant degradation of the central wavelength and spectral width was observed, as shown in Fig. 3d and Fig. S7c (Supporting Information), proving its long-term stability.

Additionally, NLP LS can also be successfully generated in the same Ta_2_PdS_6_-based fiber laser by adjusting the PC tilt angle. The spectral variation of the NLP state (Fig. 3e) demonstrates a more pronounced spectral broadening of the NLP state than the CS state. Figure 3e also shows the detailed characteristics of the NLP state output spectrum at a maximum pumping power of 830 mW, with the spectrum centered at 1562.1 nm and a 3 dB bandwidth of 27.5 nm. The average output power of NLP state operation varies with the absorbed pump power, as shown in Fig. S8a (Supporting Information). The pump power threshold was 450 mW, and the effective power operating range was 450−830 mW. The maximum average output power of the NLP state was 45 mW with a corresponding slope efficiency of 6.1%. The typical pulse train was obtained at pump powers of 450−830 mW, corresponding to a round trip time of approximately 70.9 ns (Fig. S8b, Supporting Information), the same as the CS mode due to the identical laser configuration. Figure 3f shows the autocorrelation trace data for a pump power of 830 mW. It is worth noting that the base width and spike width change with the pump power of NLP state operation. As the pump power increases, the spike width decreases from 151 to 98 fs, and the base width increases from 14.9 to 21.4 ps when pump power increases from 450 to 830 mW (Fig. 3g), which is a typical characteristic of NLP state operation. The RF spectra of the NLP state (Fig. S8d, Supporting Information) show the sharp peak of the fundamental repetition rate of 14.1 MHz and the SNR of ~55 dB, indicating high stability of NLP operation. No significant degradation of the central wavelength and spectral width was observed in the measured spectral properties (Fig. 3h and S8e, Supporting Information) under long-time NLP state operation, indicating that the laser has the potential for long-term stable operation.

**Fig. 4:**
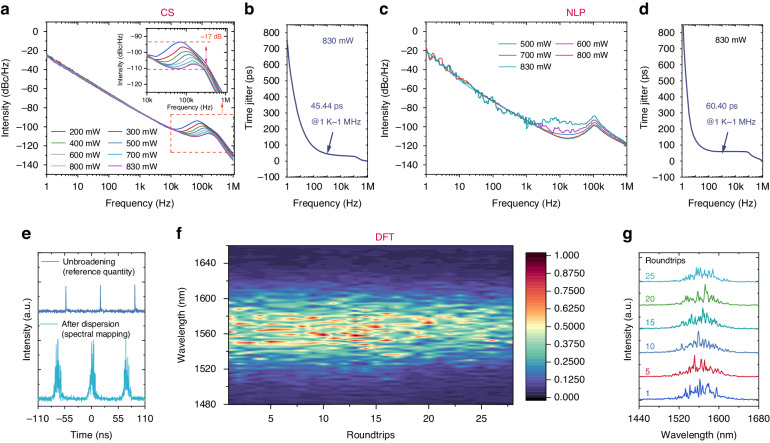
Noise performance of the CS and NLP lasers based on Ta_2_PdS_6_. **a** The phase noise characteristics of the CS state laser. **b** The timing jitter of CS under pump power of 830 mW. **c** The phase noise characteristics of the NLP state laser. **d** The timing jitter of NLP under pump power of 830 mW. **e** Typical experimental results based on the DFT technique. **f** DFT recording of single-shot spectra over 25 consecutive round trips. **g** The six typical frames of NLP spectra based on the DFT technique

The threshold of stable CS state is 200 mW, while the NLP state is 450 mW. This is difference also in the noise performance between CS and NLP states. The phase noise of the CS state decreases as the frequency increases monotonically from 1 Hz to 10 KHz, as demonstrated in Fig. 4a. The timing jitter of the CS state is calculated to be about 45.44 ps in the frequency range of 1 Hz−1 MHz at the pump power of 830 mW (Fig. 4b), further demonstrating the good stability of the laser pulse. The power spectral density curve of phase noise of NLP state appeared only when the pump power reached 500 mW (Fig. 4c), which can be attributed to the characteristics of NLP, i.e., a cluster formed by a series of sub-pulses with randomly distributed amplitudes and durations. The timing jitter is calculated to be about 60.40 ps at the frequency range of 1 Hz−1 MHz (Fig. 4d), which is inferior to that in the CS state. To further characterize the variation of the NLP state, the dispersive Fourier transformation (DFT) technique was employed (see Methods and Fig. S5d in Supporting Information). Typical experimental results based on the DFT technique (Fig. 4e) show that the spectral mapping of the stretched NLP is different at each instant. In this experiment, the real-time DFT spectral measurements were analyzed to provide a spectral characterization of the dynamics of the NLP state, as shown in Fig. 4f. Compared to spectrometer measurements, the flanks of NLP spectra measured by the DFT technique vary from shot to shot, but the bandwidth is essentially constant. For additional information, six frames of a typical shot-to-shot spectral trajectory are shown in Fig. 4g, which clearly illustrates the evolution of the NLP spectra. The strongest peaks of the spectrum alternate at center wavelengths and the sideband evolution is muddled. The results further demonstrate the wave packet nature of the NLP state, whose internal structure is a series of pulses with randomly distributed amplitudes and durations.

### “0−1”- LSAC in ultrafast fiber lasers by polarization control

**Fig. 5:**
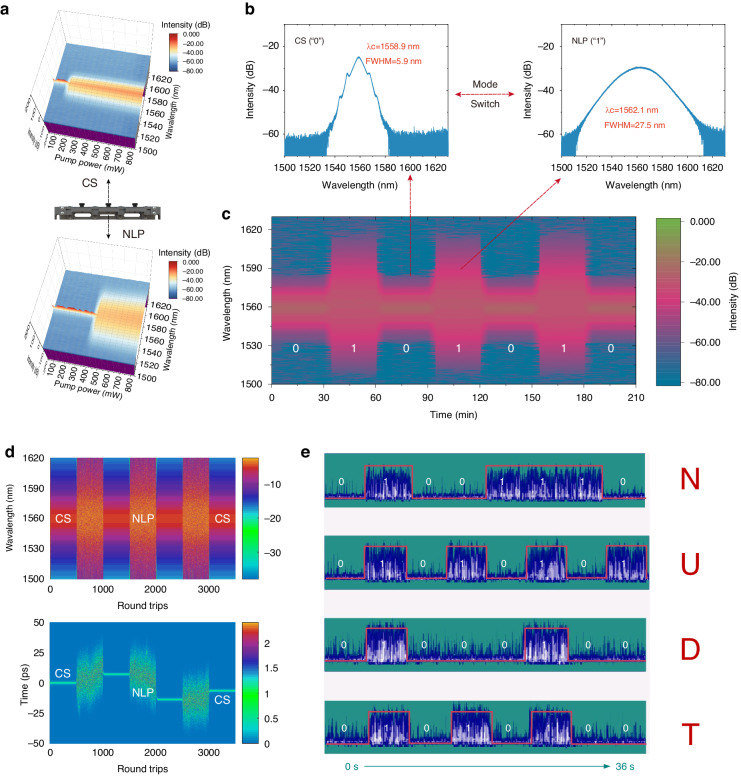
Ultrafast LSAC in the fiber laser based on Ta_2_PdS_6_ “0−1”-switcher. **a** Ultrafast fiber laser conversion demonstration. **b** The soliton and NLP spectra. **c** The output spectrum of a Ta_2_PdS_6_-based ultrafast fiber laser for a continuous conversion operation. **d** Spectral and temporal evolutions of CS-NLP LSs conversion across 3500 consecutive round trips. **e** A“NUDT” is encoded digitally

By adjusting the PC, we successfully achieved a stable CS LS output in the pump power of 200−830 mW and a stable NLP LS output in the pump power of 450-830 mW, as shown in Fig. 5a. In the pump power range of 450 mW to 830 mW, it is possible to achieve switching between the two LSs (CS-NLP) at a constant pump power just by adjusting the tilt angles of PC (refer to Supplementary Video). The spectra of soliton and NLP at a maximum pump power of 830 mW are illustrated in Fig. 5b. The CS operation was located at 1558.9 nm with a 3 dB spectral width of 5.9 nm while the NLP at 1562.1 nm with 3 dB spectrum bandwidth of 27.5 nm. Their state stability further strengthens the reliability of conversion between the two photonic states of the Ta_2_PdS_6_-based ultrafast fiber laser. We recorded changes in the output spectrum of the laser for a continuous conversion operation (adjust the tilt angles of PC) over a period of 3.5 h (seven operations, each run lasting 0.5 h), as demonstrated in Fig. 5c. The spectra of the two pulse states are essentially unchanged before and after conversion, as well as being very smooth over the 3.5 h running time, demonstrating the LSAC stability of the Ta_2_PdS_6_-based ultrafast fiber laser. It further illustrates the reliability of the results of the relative polarization analysis of saturable absorption. The phenomenon of conversion between NLP and other soliton states was observed in NPR fiber lasers^16,17^. However, the generation of LSAC in ultrafast lasers based on real saturable absorbers (SAs) is more compact and convenient.

The mode switch effect is attributed to the polarization-sensitive nonlinear absorption behavior, originating from the anisotropic LDMs. Numerical simulation was performed based on a generalized nonlinear Schrödinger equation (see Methods for details). Figure S9a (Supporting Information) shows the evolutionary process of the CS with the round-trip (RT) of 500, when the $\varDelta R$, and ${T}_{ns}$ are taken as 0.22 MW cm^1^, 32.2%, and 65.8%, respectively, and the dynamics of the simulated spectra and pulses match the typical CS characteristics (Figs. S10a, S10b, Supporting Information). After modulation of the nonlinear parameters of the Ta_2_PdS_6_ “0−1”-switcher by changing the PC, the $\varDelta R$, ${I}_{s}$, and ${T}_{ns}$ are set as 0.21 MW cm^1^, 31.2%, and 56.3% while other parameters remain constant. As illustrated in Fig. S9b (Supporting Information), the dynamics of the simulated spectra and pulses match the typical NLP characteristics, and the spectral profile and pulse autocorrelation trace are shown in Figs. S10c and S10d (Supporting Information). Furthermore, the Ta_2_PdS_6_ “0−1”-switcher-based regulation also causes changes in the intensity of the intracavity nonlinear effects, where the solitons split into multiple pulses due to soliton quantization effects. Consequently, the evolutionary process of the CS (“0”)-NLP (“1”) state conversion happens in the system (seven operations, each RT of 500), which is consistent with the experimental results (Fig. 5d).

The stable conversion of different pulse states in a single ultrafast fiber laser system has the potential for extended applications, such as communications coding and optical switching. The conversion of CS (“0”) and NLP (“1”) is displayed on the oscilloscope by the intensity of the pulse signal with a level difference of about 0.02 V (Fig. S11a, and Supplementary Video). The application of digital coding can be realized by receiving the optical signal through the photoelectric detector. The optical binary codes of NUDT (01001110010101010100010001010100, an abbreviation from the National University of Defense Technology) can be obtained by the Ta_2_PdS_6_ “0-1”-switcher, as shown in Fig. 5e. In this work, the adjustment of the PC is manually controlled, and the maximum switching frequency that can be achieved is in the order of a few Hz. By time domain analysis, the time interval between the two luminescent state transitions is about 42 μs, as shown in Fig. S11b (Supporting Information). As a result, It can be expected that the dual-state switching frequency can reach the kHz magnitude by introducing a mechanically tunable motorized device. Combined with the steady conversion and stable operation of the CS and NLP states, tunable photonics devices based on anisotropic materials are expected to offer new prospects in ultrafast laser applications.

## Discussion

In summary, the quasi-1D layered Ta_2_PdS_6_ with anisotropic optical absorption was investigated to modulate the property of nonlinear absorption as SA. Both steady-state CS (“0”) and NLP (“1”) pulsed laser modes were achieved by altering the polarization of light. The CS state produces an ultrashort pulse with a pulse width of 697 fs and a pulse energy of 2.62 nJ. NLP state operation yielded pulses with spike widths as short as 98 fs. Additionally, the noise features and long-term stability for both two modes have been systematically investigated. A Ta_2_PdS_6_ “0-1”-switcher was further demonstrated and explained by numerical simulation. LSAC laser was used as a codable light source to demonstrate their application for digital coding. The findings demonstrate that anisotropic quasi-1D layered materials are an efficient optical modulator for LSAC, which will pave the way for developing tunable ultrafast photonic devices.

## Materials and methods

### Synthesis of Ta_2_PdS_6_ Single Crystals

By using the iodide as the transport agent, the high-quality single crystals of Ta_2_PdS_6_ were synthesized via a CVT method. The 0.3 g of high purity Ta, Pd, and S, and 120 mg of iodide were sealed in a 20 cm-long vacuum quartz tube under a vacuum of 10^−6^ Torr and placed in a two-zone furnace. The source end is then slowly heated to 875 °C and the other end is kept at 730 °C to maintain a relatively low temperature with the source end. The heating process held for 168 h. The furnace is then naturally cooled to room temperature. Shiny silver needle-like single crystals of Ta_2_PdS_6_ are obtained at the cold end of the tube.

### Characterizations

ARPES data were gathered by a DA30L analyzer with energy resolution and angular resolution set to 20 meV and 0.2°, respectively. XPS measurement was carried out on a spectrometer outfitted with a monochromatic Al Kα X-ray source (1486.6 eV) that operates at 100 W. Raman data were measured by a Raman spectrometer (Lab RAM HR 800) with a laser wavelength of 532 nm. STEM images were obtained on the instruments (Thermo Scientific, Themis Z) operating at 300 kV. UV-vis-NIR spectrophotometry (UV-3600 Plus, SHIMADZU) was performed to study the absorption property of the Ta_2_PdS_6_.

### Theoretically simulated of polarized optical absorption

The following formula was employed to simulate the direction-dependent optical properties^43^:

2 $$\alpha (\omega )=\sqrt{2}\omega {\left[\sqrt{{\varepsilon }_{1}^{2}+{\varepsilon }_{2}^{2}}-{\varepsilon }_{1}\right]}^{\frac{1}{2}}$$

where the *ε*_1_(*ω*) and *ε*_2_(*ω*) represent the real and imaginary parts of the dielectric function, respectively. The imaginary part *ε*_2_(*ω*) of the dielectric function can be obtained by evaluating the momentum matrix elements between the occupied and unoccupied states using the following expression:

3 $${\varepsilon }_{2}(\omega )=\frac{4{\pi }^{2}{e}^{2}}{\varOmega }\mathop{\mathrm{lim}}\limits_{q\to 0}\frac{1}{{q}^{2}}\sum _{c:\nu :k}2{\omega }_{k}\delta ({\varepsilon }_{ck}-{\varepsilon }_{ck}-\omega )\times \langle {u}_{ck+{e}_{\alpha }q}|{u}_{\nu k}\rangle {\langle {u}_{ck+{e}_{\beta }q}|{u}_{\nu k}\rangle }^{\ast }$$

where Ω represents the volume, *α*, and *β* are the Cartesian components, *e*_*α*,_ and *e*_*β*_ represent the unit vectors, *v*, and *c* correspond to the matrix elements of the transition from the valence band state (*u*_*vk*_) to the conduction band state (*u*_*ck*_), *ε*_*ck*_ and *ε*_*vk*_ signify for the energy of the conduction and valence band, respectively. The real part *ε*_1_(*ω*) of the dielectric function can be derived from the imaginary part using the Kramer-Kronig relationship, expressed as:

4 $${\varepsilon }_{1}(\omega )=1+\frac{2}{\pi }P{\int}_{0}^{\infty }\frac{{\varepsilon }_{2}^{\alpha \beta }(\omega ^{\prime} )\omega ^{\prime}}{\omega ^{\prime 2}-{\omega}^{2}+i\eta }d\omega ^{\prime}$$

where *P* represents the principal value of the integral.

### Experimental setup for polarization absorption of Ta_2_PdS_6_

The light source was a 1.56 μm homemade laser, and the emitted light was passed through a collimator and then through a polarized beam splitter. The beam was then passed through a half-wave plate and onto the Ta_2_PdS_6_ sample. The polarization state of the light was changed by turning the half-wave plate and the change in transmitted power was measured with a power meter (PM).

### Experimental setup for the measurement of the nonlinear polarization optical response

The structure of the measurement system consists of a homemade 1.56 μm seed laser (pulse width: 260 fs; repetition frequency: 13.0 MHz) and an erbium-doped fiber amplifier. The laser beam is divided into two paths by a 50:50 optical coupler (OC): contrasting path and test path. In the contrasting path, the power is measured using a PM 1. In the test path, the intensity of the laser interaction with the material can be varied by adjusting the amplifier pump, and the power is measured with PM 2 after the laser has passed through the sample. Furthermore, a polarization-independent isolator (ISO) and a PC are added to the optical path of measurement to simply simulate the nonlinear optical response of Ta_2_PdS_6_ “0-1”-switcher after polarization changes caused by the tilt angles of PC in the laser cavity and the adjustment position change (tilt angle with 0°, 45°, 90°, 135°, and 180°) of PC.

### Experimental setup of Ta_2_PdS_6_-based ultrafast fiber laser

The device diagram of the fiber laser is shown in Fig. S5b (Supporting Information). A laser diode (LD, maximum output power of 830 mW) with a center wavelength of 980 nm was selected as the pump light source, and the light source is coupled to the cavity through a 980/1550 wave division multiplexer (WDM). The gain medium of the fiber laser was a 2.4-m-long EDF (dispersion coefficient of 28 ps^2^·km^−1^) to achieve the inversion of particle numbers. The PC was used to adjust the polarization state in the laser cavity, and the ISO was used to control the unidirectional transmission of light. The 2:8 OC coupled 80% of the laser into the WDM and output 20% of the laser. All optical fibers were SMF (dispersion coefficient of −23 ps^2^·km^−1^). The total length of the laser cavity is about 14.6 m with a total dispersion of about −0.21 ps^2^.

### Experimental setup of the DFT

The schematic of the real-time detection system based on the DFT technique is shown in Fig. S5d (Supporting Information). The laser source is a Ta_2_PdS_6_-based ultrafast fiber laser constructed for our experiment. The laser source was tuned to the NLP output state, and the laser light output was split into two paths via an optocoupler. One path (the reference volume) is used to record the evolution of the instantaneous intensity pattern of the pulse. The signal from the other path is fed into an approximately 10.4 km long normal SMF to time-stretch the pulse (~230 ps^2^) for DFT-based spectral measurements^44^. The signals from the two paths were detected by two identical high-speed photodetectors (PD1 and PD2) and recorded on a real-time oscilloscope at a sampling rate of 5 GS s^−1^ and a bandwidth of 1 GHz. The resolution of the corresponding DFT measurements was about 0.2 ns.

### Simulation

The numerical simulation was performed to model the LS evolution dynamics based on generalized nonlinear Schrödinger equation^45^:

5 $$\frac{\partial \psi (z,t)}{\partial z}=\frac{g}{2}\left(\psi (z,t)+\frac{1}{{\varOmega }_{g}^{2}}\frac{{\partial }^{2}\psi (z,t)}{\partial {\tau }^{2}}\right)-\frac{i{\beta }_{2}}{2}\frac{{\partial }^{2}\psi (z,t)}{\partial {\tau }^{2}}+i\gamma {|\psi (z,t)|}^{2}\psi (z,t)$$

where $\psi (z,t)$, $z$, ${\beta }_{2}$, $\gamma$, and ${\varOmega }_{g}$ are the slow pulse electric field envelope, propagation coordinate, second-order dispersion, Kerr nonlinearity coefficients, and bandwidth of the gain, respectively. The gain coefficient $g$ is given by the following equation.

6 $$g(z)={g}_{0}\exp \left(\frac{-{E}_{p}}{{E}_{s}}\right)$$

7 $${E}_{p}(z)={\int dt|\psi (z,t)|}^{2}$$

where ${g}_{0}$, ${E}_{p}$, and ${E}_{s}$ are the small signal gain, pulse energy, and gain saturation energy, respectively. The Ta_2_PdS_6_-based mode-locking method can be modeled by the instantaneous nonlinear transfer function of Eq.(1). The numerical model is solved with a split-step Fourier method, and the initial input pulse is a weak Gaussian pulse. The other parameters are matched to the experimental conditions, and the specific parameters are as follows: erbium-doped fiber (EDF) = 2.4 m, single-mode optical fibers (SMF) = 12.2 m, ${E}_{s}$ = 100 pJ, ${\varOmega }_{g}$ = 30 nm, $\gamma$ = 5 W^−1^·km^−1^; ${g}_{0}$ = 2.9 m^−1^, ${\beta }_{2}$= 28 ps^2^·km^−1^ for EDF; ${g}_{0}$ = 0, ${\beta }_{2}$ = −23 ps^2^·km^−1^ for SMF.

### Supplementary information

Supporting Information for Ultrafast laser state active controlling based on anisotropic quasi-1D material

Supplementary Video

## Data Availability

The data that support the plots within this paper and other findings of this study are available within this article and its Supplementary Information file, and are also available from the corresponding author upon request.

## Code availability

The code that supports the findings of this study is available from the corresponding author on request.
