# Supplementary material for: Ultrafast laser state active controlling based on anisotropic quasi-1D material
Source: Light Sci Appl. 2024 Apr 7;13:81. doi: 10.1038/s41377-024-01423-3 (PMC11251271; doi:10.1038/s41377-024-01423-3)
Supplement: Supplementary file 1 — Supporting Information for Ultrafast laser state active controlling based on anisotropic quasi-1D material [file 41377_2024_1423_MOESM1_ESM.docx]

**Supporting Information** **for Ultrafast laser state** **active controlling based on** **anisotropic quasi-1D material**

Zixin Yang^1,6,#^, Qiang Yu^1,2,#^, Jian Wu^1,*^, Haiqin Deng^1^, Yan Zhang^2^, Wenchao Wang^3^, Tianhao Xian^4^, Luyi Huang^2^, Junrong Zhang^2^, Shuai Yuan^5^, Jinyong Leng^1,6^, Li Zhan^4^, Zongfu Jiang^1,6^, Junyong Wang^2,*^, Kai Zhang^2,*^, Pu Zhou^1,*^

*^1^**College of Advanced Interdisciplinary Studies,* *National University of Defense Technology, Changsha, 410073, China*

*^2^i-Lab & Key Laboratory of Nanodevices and Applications & Key Laboratory of Nanophotonic Materials and Devices, Suzhou Institute of Nano-Tech and Nano-Bionics, Chinese Academy of Sciences, Suzhou, 215123, China*

*^3^School of Applied and Engineering Physics, Cornell University, Ithaca, New York 14853, USA*

*^4^State Key Laboratory of Advanced Optical Communication Systems and Networks, School of Physics and Astronomy, Shanghai Jiao Tong University, Shanghai 200240, China*

*^5^Shanghai Key Lab of Modern Optical System, University of Shanghai for Science and Technology, Shanghai 200093, China*

*^6^Nanhu Laser Laboratory, National University of Defense Technology, Changsha, 410073, China*

*^#^These authors contributed equally: Zixin Yang, Qiang Yu*

*E-mail:* *wujian15203@163.com;* *jywang2022@sinano.ac.cn;* [*kzhang2015@sinano.ac.cn*](mailto:kzhang2015@sinano.ac.cn)*; zhoupu203@163.com*

1. **Characterization of the quasi-1D Ta_2_PdS_6_**
2. **U****ltrafast laser setups and polarization absorption mechanism diagram**
3. **O****utput property of state active controlling**
4. **Characterization of the quasi-1D Ta_2_PdS_6_**


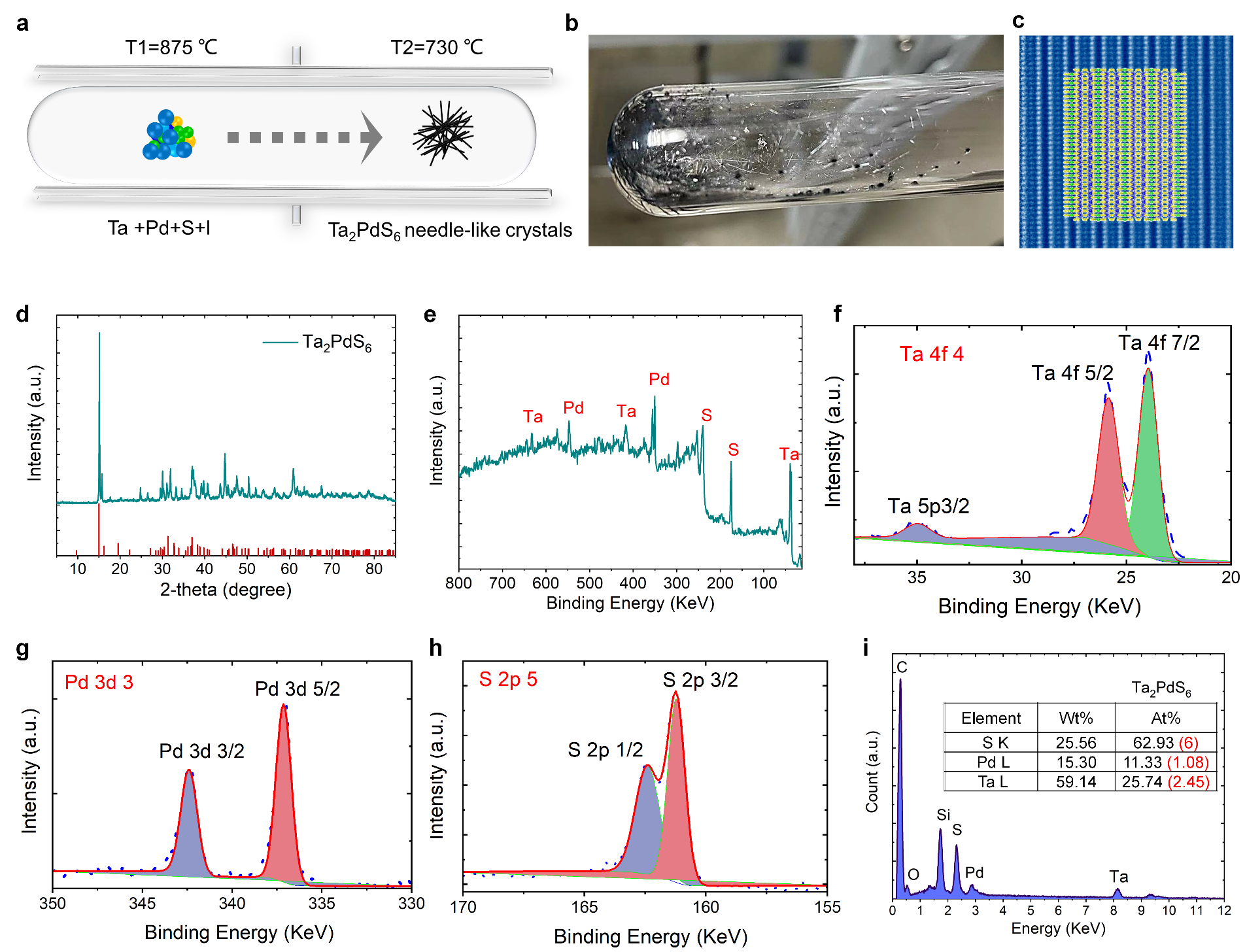


**Fig. S1. Synthesis and characterizations of Ta_2_PdS_6_.** **a** Schematic diagram of the growth of a Ta_2_PdS_6_ single crystal. **b** The physical image of the as-prepared Ta_2_PdS_6_. **c** High-magnification TEM image of quasi-1D Ta_2_PdS_6_. **d** XRD patterns of Ta_2_PdS_6_. **e** XPS spectrum of Ta_2_PdS_6_. **f-h** The high-resolution XPS spectra of the Ta (f), Pd (g), and S (h), respectively. **i** EDS profiles of Ta_2_PdS_6_.

The Ta_2_PdS_6_ crystal phase was investigated using the X-ray diffraction (XRD) technique (Fig. S1d). The signals of Ta, Pd, and S elements were observed by X-ray photoelectron spectroscopy (XPS) measurement with an atomic ratio of ∼2:1:6 (Fig. S1e-h), quantitively confirming its high quality. The dispersive spectrometer (EDS) test and the corresponding elemental mappings of Ta, Pd, and S further corroborate the components (Fig. S1i).


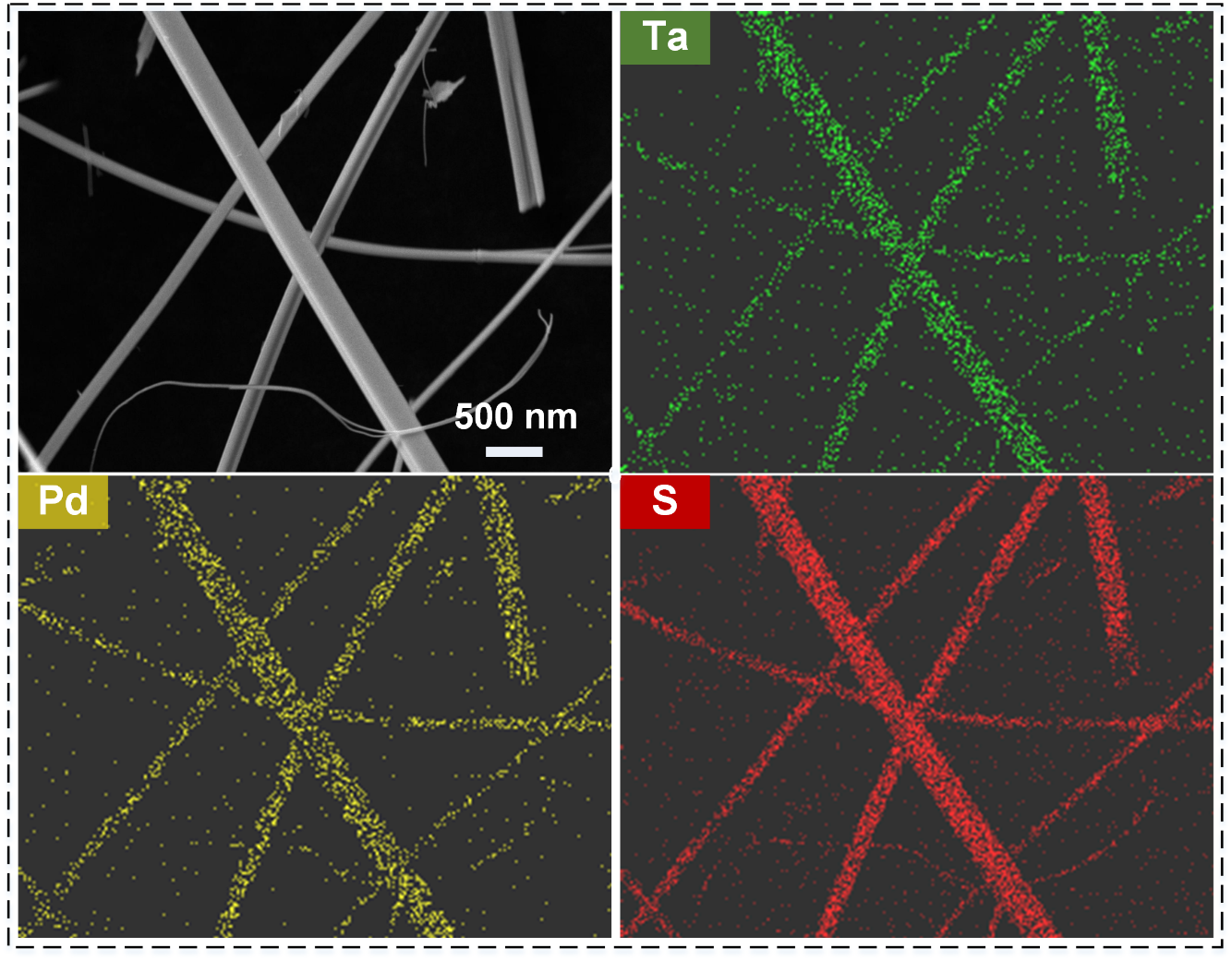


**Fig. S2. The SEM image of bulk Ta_2_PdS_6_ and corresponding EDS elemental mappings of Ta, Pd, and S for the crystal, respectively**.

The scanning electron microscopy (SEM) image of Ta_2_PdS_6_ (Fig. S2) clearly shows its needle-like properties due to the long axis of the single crystal along the *b*-axis.


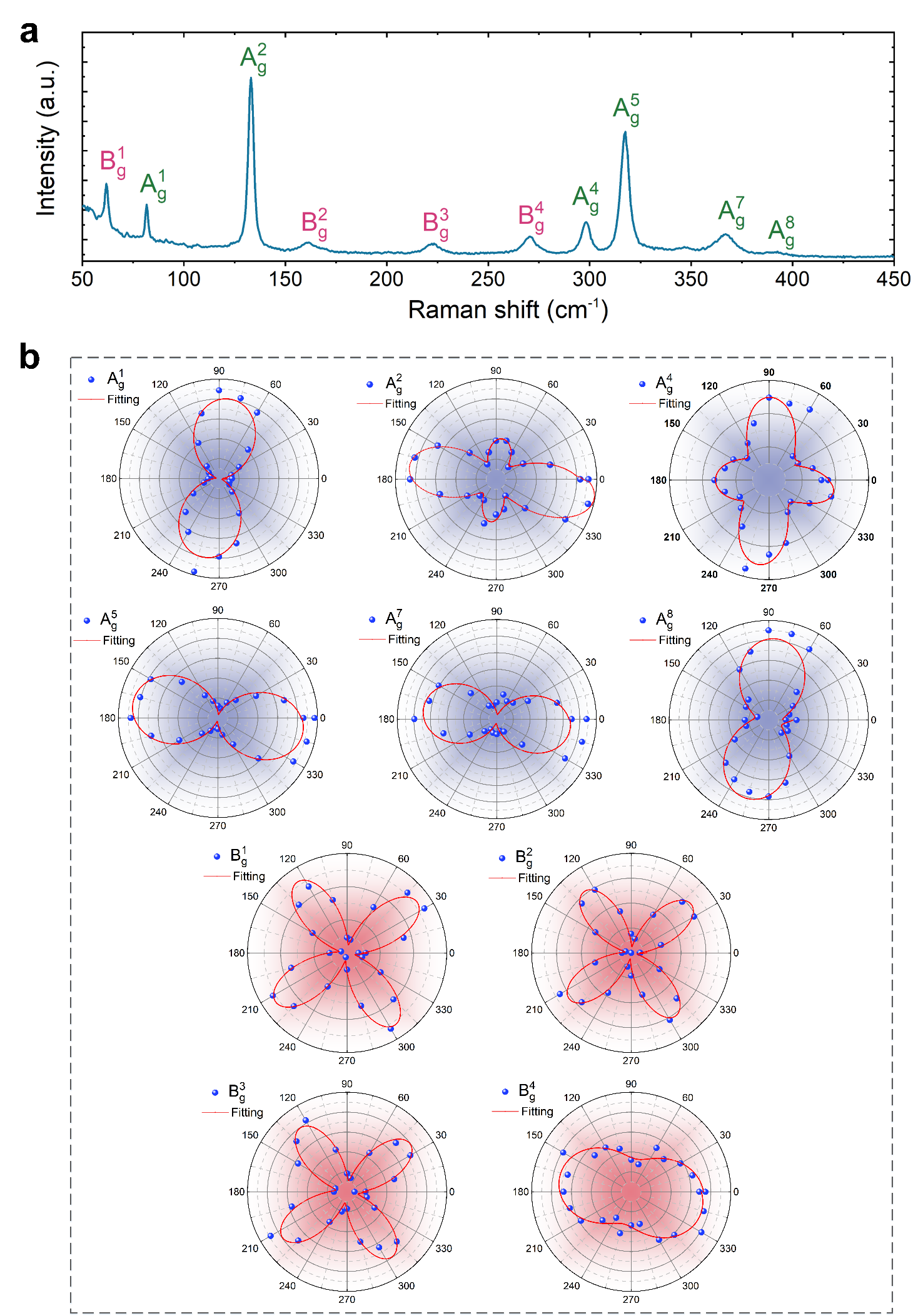


**Fig. S3. Raman characterization. a** The Raman spectrum of Ta_2_PdS_6_. **b** **The evolution of the Raman intensities for $A_{g}$ and $B_{g}$ modes under different polarization states.**

The phonon vibration of the quasi-1D Ta_2_PdS_6_ is studied by polarized Raman spectroscopy. The Ta_2_PdS_6_ crystal structure belongs to the point group C_2h_. The active modes can be described by the Raman tensors in the group theory analysis as follows：

$A_{g}=\left[\begin{matrix} a & 0 & d \\ 0 & b & 0 \\ d & 0 & c \end{matrix}\right],B_{g}=\left[\begin{matrix} 0 & e & 0 \\ e & 0 & f \\ 0 & f & 0 \end{matrix}\right]$ (1)

The theoretical analysis of the group shows that there are two main broad categories of Raman vibrational modes for Ta_2_PdS_6_, that of $A_{g}$ and $B_{g}$, which are supported by the experimental results (Fig. S3a) for the $A_{g}^{1}$, $A_{g}^{2}$, $A_{g}^{4}$, $A_{g}^{5}$, $A_{g}^{7}$, $A_{g}^{8}$, $B_{g}^{1}$, $B_{g}^{2}$, $B_{g}^{3}$, and $B_{g}^{4}$ modes. The Raman modes mainly concentrated at wave numbers of 50-400 cm^-1^. They corresponding to the Raman peak are 81.6 cm^-1^, 133.1 cm^-1^, 298.2 cm^-1^, 317.6 cm^-1^, 366.9 cm^-1^, 391.4 cm^-1^, 61.7 cm^-1^, 161.1 cm^-1^, 222.3 cm^-1^, 270.2 cm^-1^, respectively. Angle-resolved polarized Raman spectra can provide information on the vibrational symmetry to probe its anisotropic structure. The polarized Raman spectra of the Ta_2_PdS_6_ indicate polar plots of the Raman intensity corresponding to the typical $A_{g}$ and $B_{g}$ vibrational modes, as shown in Fig. S3b. The experimental data match well with the fitted curves, and different types of Raman vibration models show different degrees of anisotropy, further indicating the strong anisotropy of the quasi-1D Ta_2_PdS_6_.

**
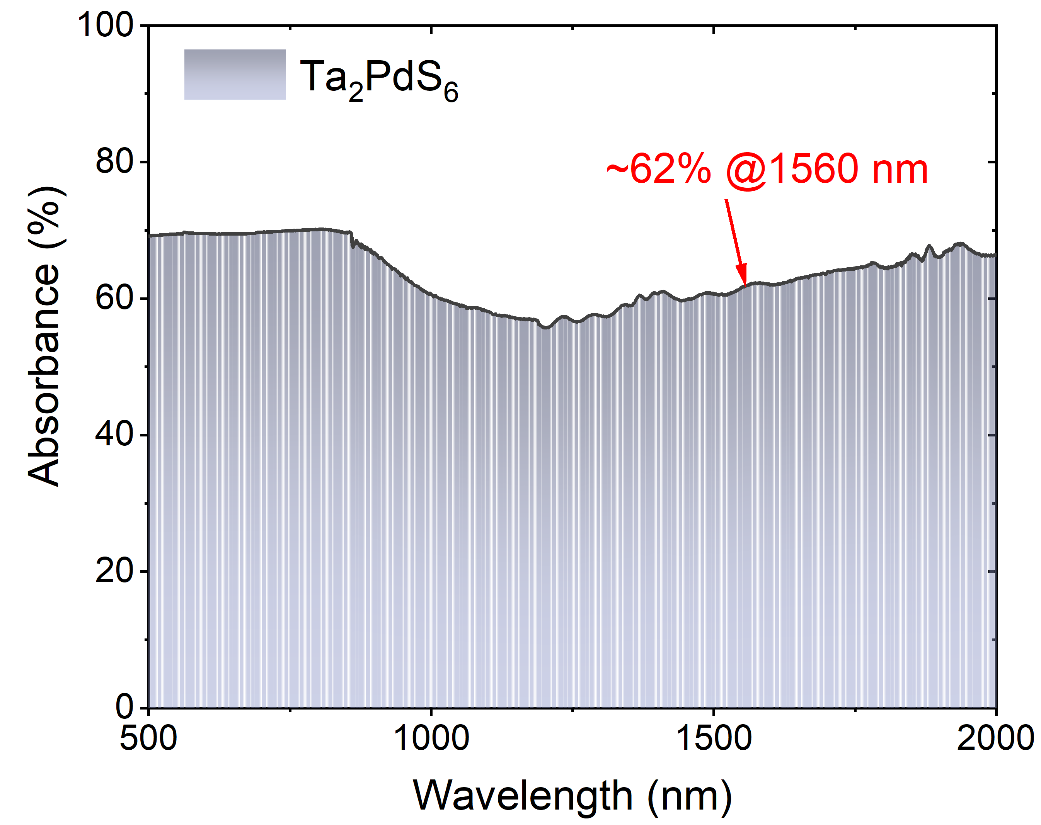
**

**Fig. S4. The UV-vis-NIR absorption spectrum of layered Ta_2_PdS_6_.**

1. **Ultrafast laser setups and polarization absorption mechanism diagram**

**
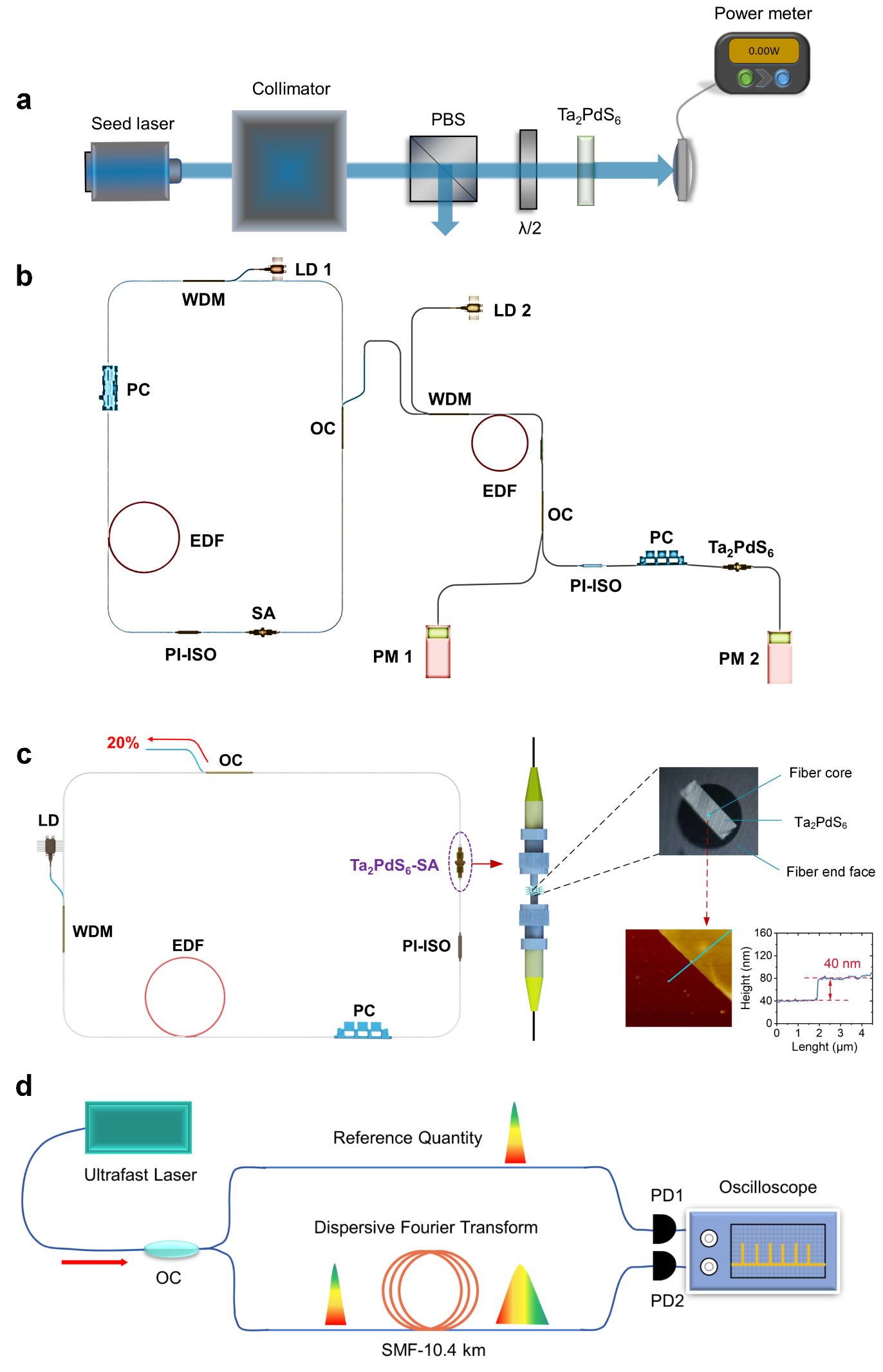
**

**Fig. S5.** **Experimental setup. a** Experimental setup of the polarization-dependent absorption for Ta_2_PdS_6_. **b** the experimental setup for measuring the polarization-resolved nonlinear optical response. **c** [Left](javascript:;): the experimental configuration of the ultrafast ﬁber laser; [Right](javascript:;): the morphology of the Ta_2_PdS_6_ device. **d** Schematic of the real-time detection system based on the DFT technique.

**
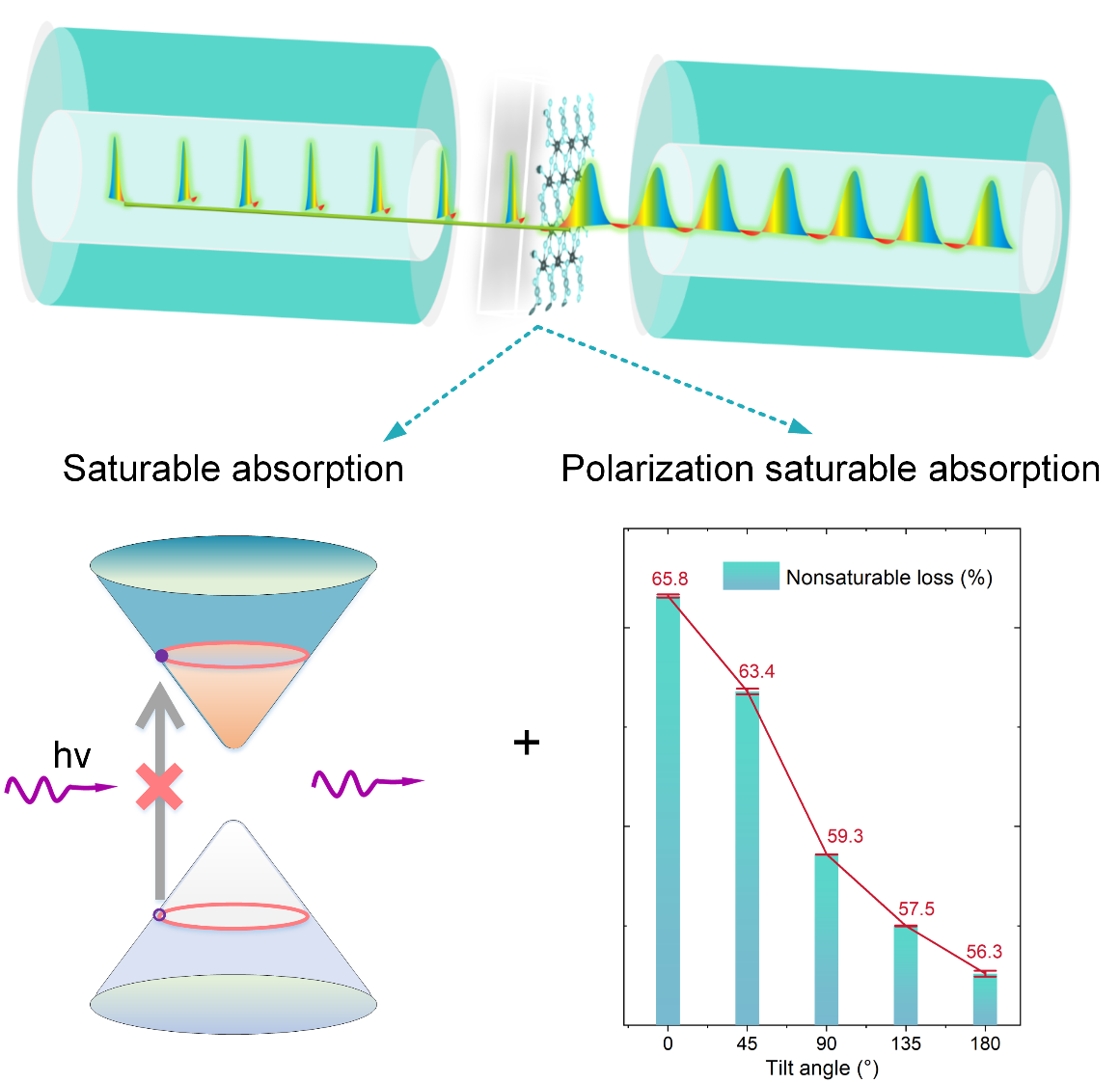
**

**Fig. S6. Schematic of saturable absorption and polarized optical response of the Ta_2_PdS_6_.**

The schematic of saturable absorption and polarized optical response of the Ta_2_PdS_6_ is shown in Fig. S6. For quasi-1D Ta_2_PdS_6_-based photonics devices, its saturable absorption mechanism can be explained by the Pauli blocking principle. First, when the incident photon energy is over the bandgap value of quasi-1D Ta_2_PdS_6_, the electrons can be excited into the conduction band and rapidly cooled to form a thermal Fermi-Dirac distribution. Under these conditions, the newly formed electron-hole pairs block the otherwise potential interband optical transitions near the Fermi energy. As a result of phonon scattering, the hot carriers cool further and the electrons and holes recombine to reach an equilibrium distribution. However, as the light intensity continues to increase, the number of photocarriers instantly increases and fills the energy states near the conduction and valence band edges. According to the Pauli blocking principle, light absorption is blocked and photons can transparently pass through quasi-1D Ta_2_PdS_6_ without being absorbed. After a relative comparative analysis, the results show that quasi-1D Ta_2_PdS_6_ has a polarization-dependent saturable absorption property, and its nonlinear absorption parameters can be altered by changing the polarization state of the incident light with identical absorber materials. The nonsaturable loss shows a significant variation trend with a maximum saturation absorption loss of about 65.8% at the tilt angles of 0° and a minimum saturation loss of about 56.3% at the tilt angles of 180°. The results demonstrate that the anisotropic quasi-1D Ta_2_PdS_6_ has an additional variable factor through the flip of PC, which is essential for the artificial regulation of the nonlinear optical parameters of Ta_2_PdS_6_.

1. **Output property of state active controlling**

**
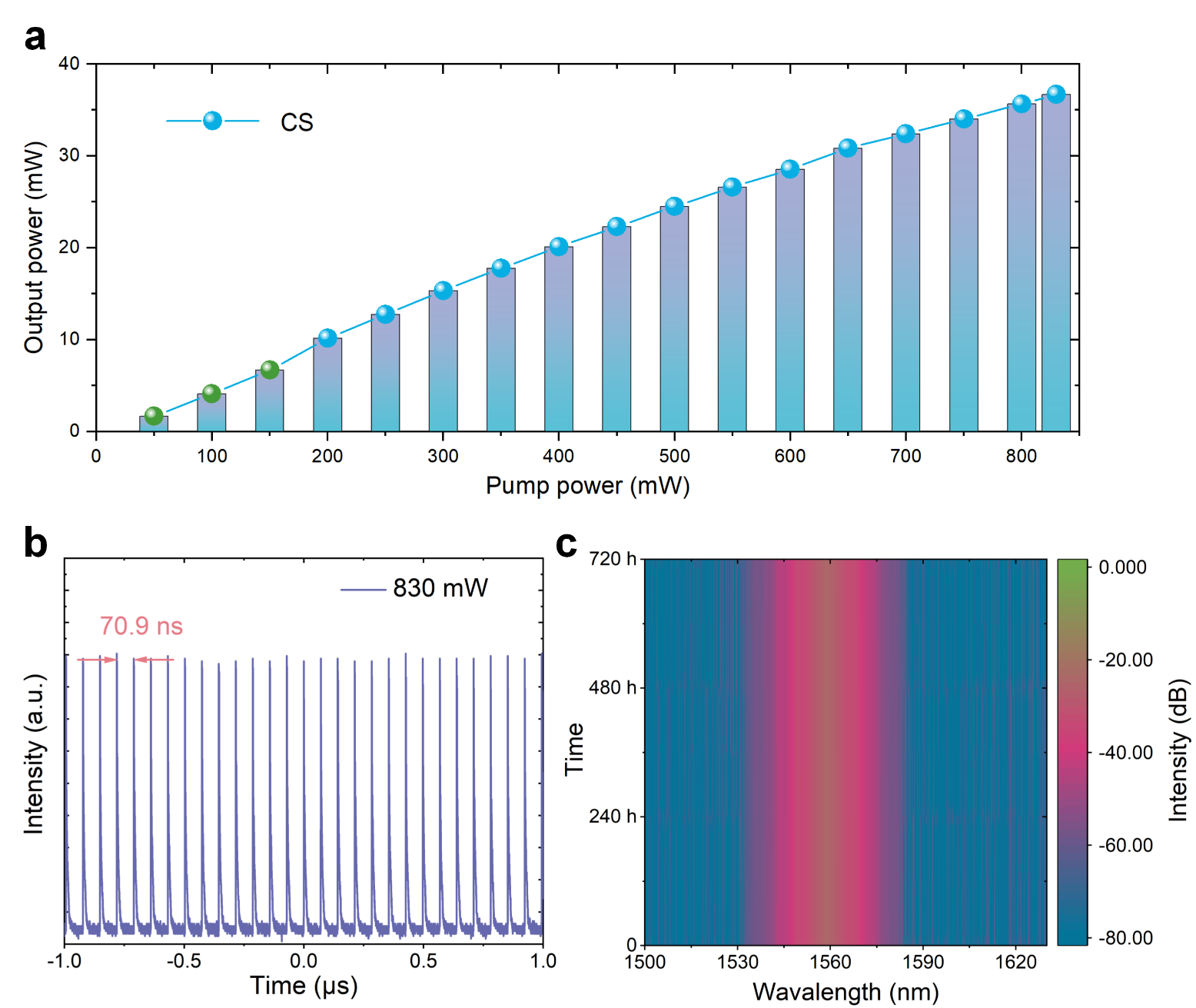
**

**Fig. S7. Laser output characteristics of CS LS.** **a** The average output powers of CS under various pump powers. **b** Pulse train under pump power of 830 mW. **c** Spectra of long-term stable operation over 720 hours of CS LS.


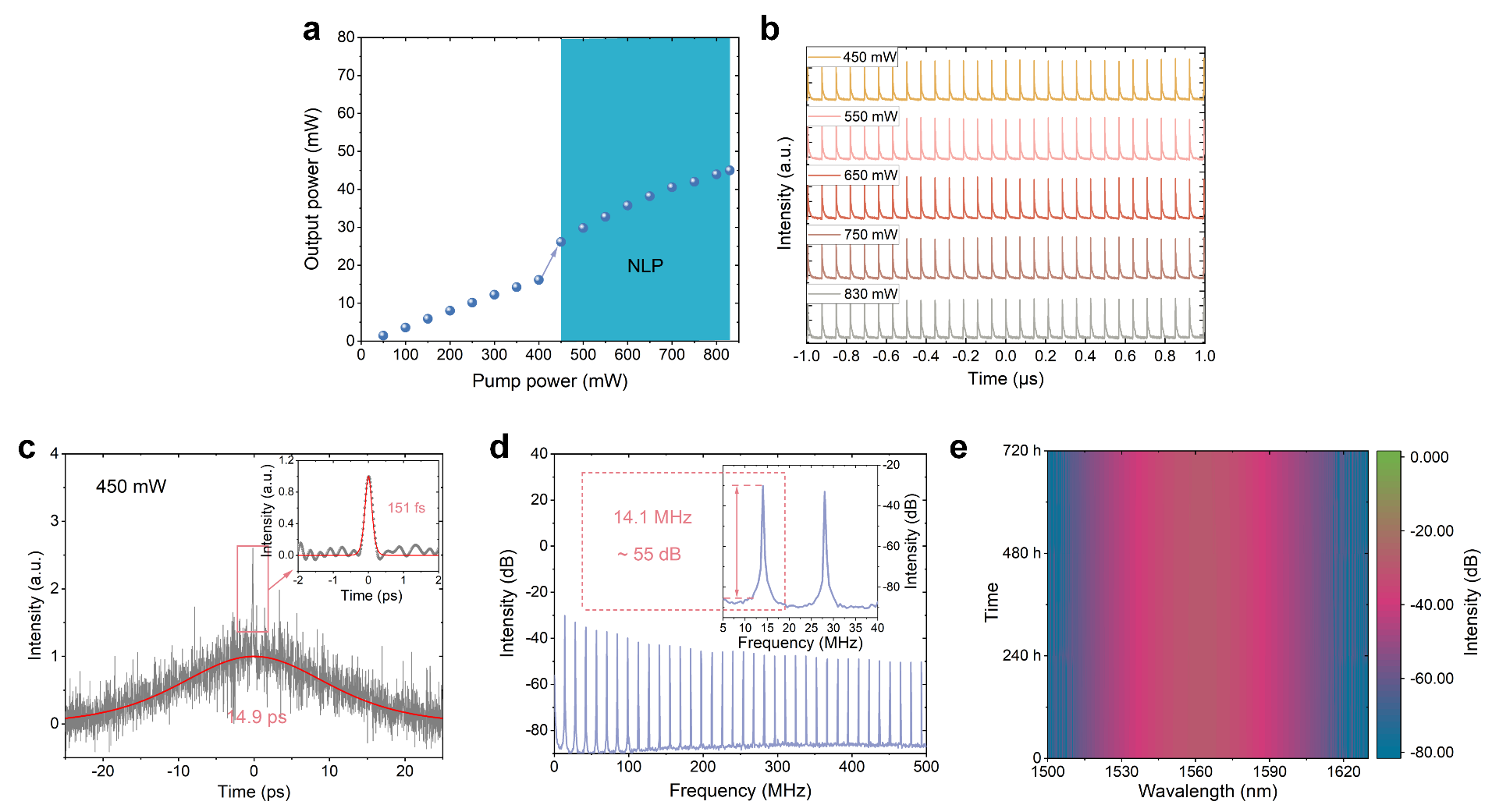


**Fig. S8. Laser output characteristics of NLP LS. a** Optical spectrum of NLP at 830 mW. **b** Pulse sequences of NLP at different pump powers. **c** Autocorrelation trace of NLP state at 450 mW. **d** RF spectrum. **e** The timing jitter of NLP LS under pump power of 830 mW. **f** DFT recording of single-shot spectra over 25 consecutive round trips. **g** Spectra of long-term stable operation over 720 hours of NLP LS. **h** The central wavelength and 3 dB bandwidth of spectra acquired at different hours.


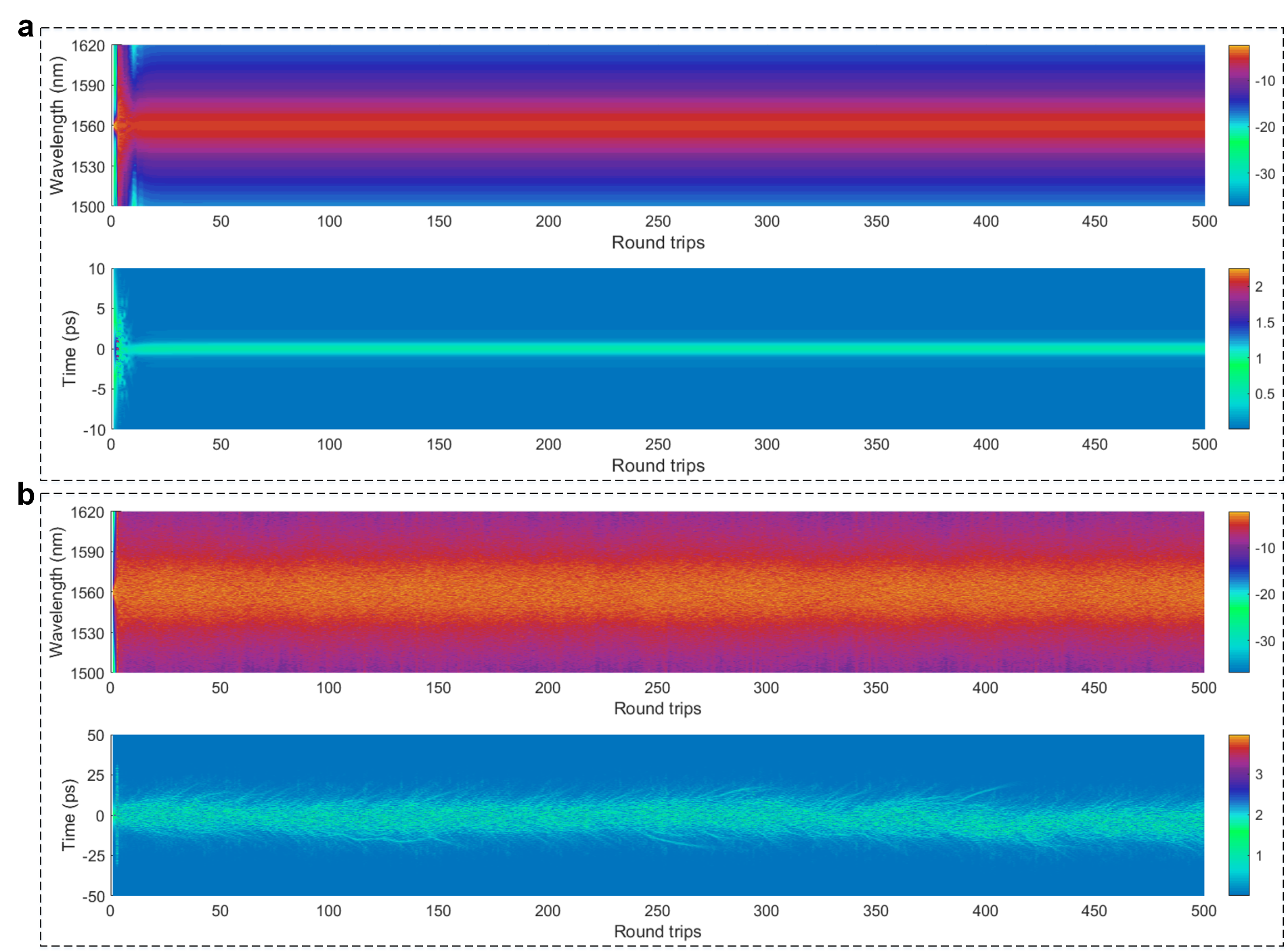


**Fig. S9. Numerical simulation results of dynamics**. **a** Spectral and temporal evolutions in the CS LS in 500 consecutive RT. **b** Spectral and temporal evolutions in the NLP LS in 500 consecutive RT.

**
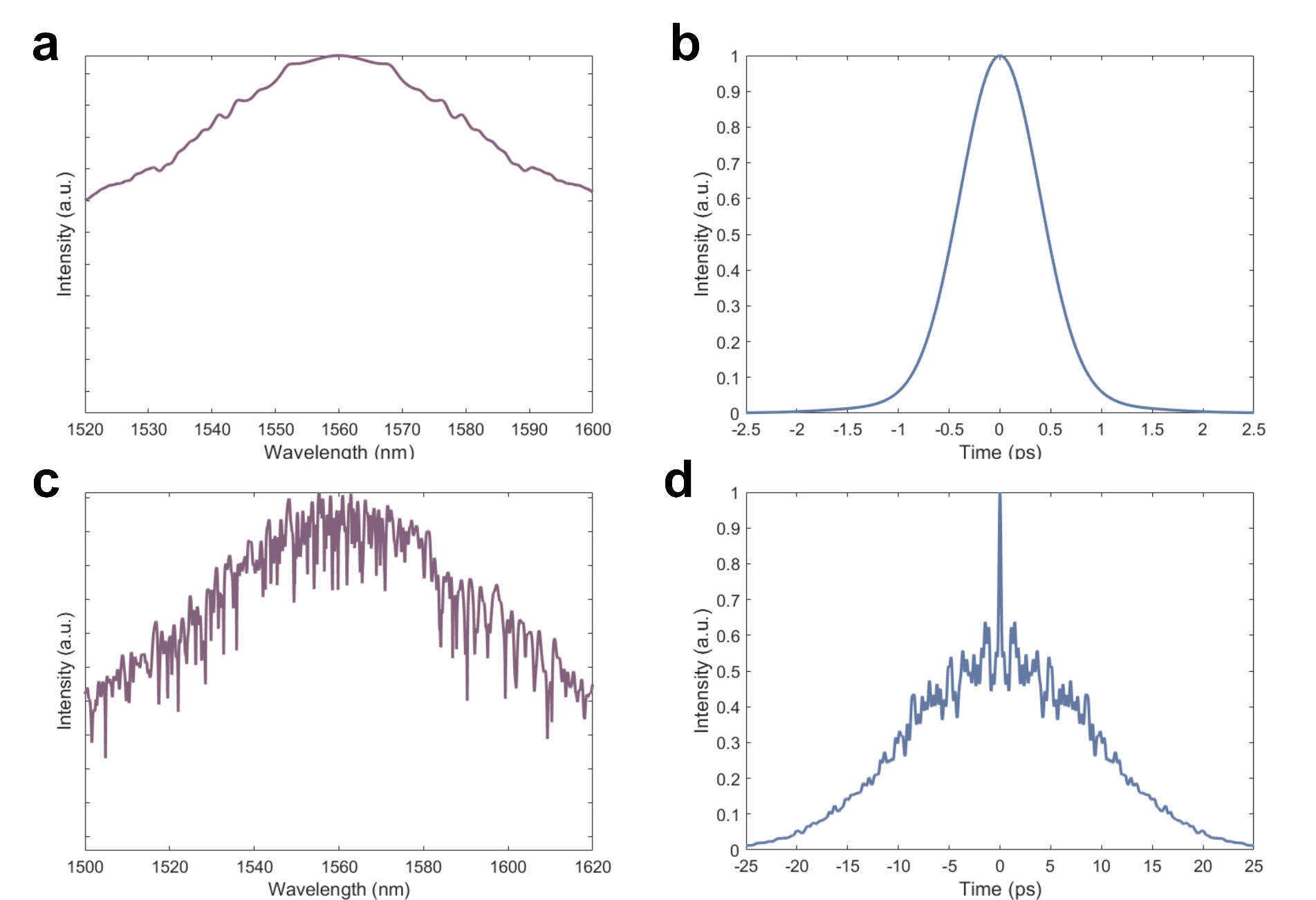
**

**Fig. S10. Numerical simulation results of dynamics with RT = 500.** **a** Spectral profile of CS. **b** Pulse autocorrelation trace of CS. **c** Spectral profile of NLP. **d** Pulse autocorrelation trace of NLP.


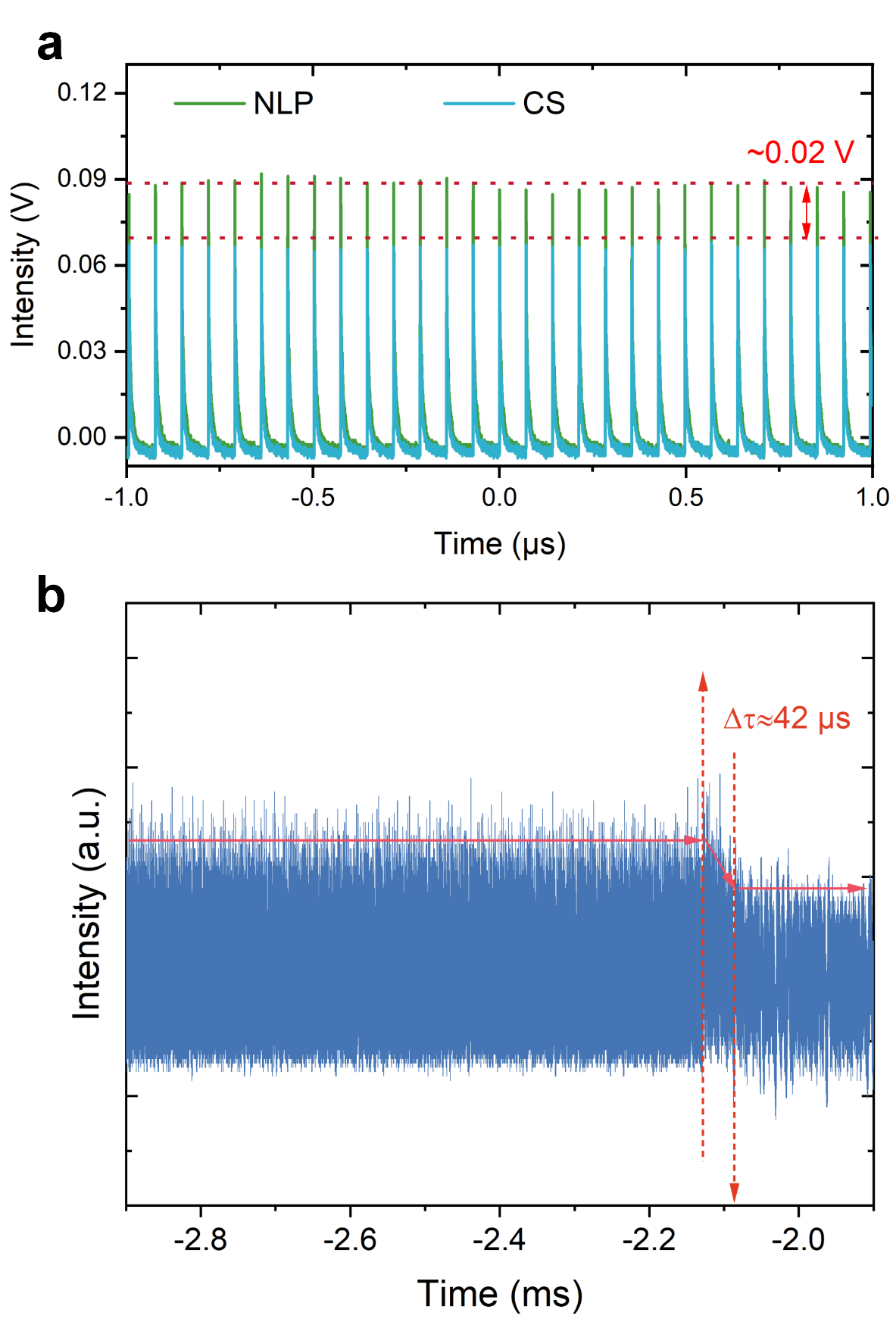


**Fig. S11. Characterization of LSs controlling. a** Pulse intensities of different LSs. **b** Time domain analysis plot for LSAC.
